# Integrated growth assessment in the first 1000 d of life: an interdisciplinary conceptual framework

**DOI:** 10.1017/S1368980023000940

**Published:** 2023-08

**Authors:** Sanja Nel, Robert C Pattinson, Valerie Vannevel, Ute D Feucht, Helen Mulol, Friede AM Wenhold

**Affiliations:** 1 Department of Human Nutrition, University of Pretoria, Pretoria 0002, South Africa; 2 Research Centre for Maternal, Fetal, Newborn & Child Health Care Strategies, University of Pretoria, Pretoria, South Africa; 3 Maternal and Infant Health Care Strategies Unit, South African Medical Research Council (SAMRC), Pretoria, South Africa; 4 Department of Obstetrics and Gynaecology, University of Pretoria, Pretoria, South Africa; 5 Department of Paediatrics, University of Pretoria, Pretoria, South Africa; 6 Tshwane District Health Services, Gauteng Department of Health, Pretoria, South Africa

**Keywords:** First 1000 d, Growth monitoring, Foetal growth restriction, Doppler, Infant growth, Interdisciplinary care, Continuity of care

## Abstract

**Objectives:**

Prenatal growth affects short- and long-term morbidity, mortality and growth, yet communication between prenatal and postnatal healthcare teams is often minimal. This paper aims to develop an integrated, interdisciplinary framework for foetal/infant growth assessment, contributing to the continuity of care across the first 1000 d of life.

**Design:**

A multidisciplinary think-tank met regularly over many months to share and debate their practice and research experience related to foetal/infant growth assessment. Participants’ personal practice and knowledge were verified against and supplemented by published research.

**Setting:**

Online and in-person brainstorming sessions of growth assessment practices that are feasible and valuable in resource-limited, low- and middle-income country (LMIC) settings.

**Participants:**

A group of obstetricians, paediatricians, dietitians/nutritionists and a statistician.

**Results:**

Numerous measurements, indices and indicators were identified for growth assessment in the first 1000 d. Relationships between foetal, neonatal and infant measurements were elucidated and integrated into an interdisciplinary framework. Practices relevant to LMIC were then highlighted: antenatal Doppler screening, comprehensive and accurate birth anthropometry (including proportionality of weight, length and head circumference), placenta weighing and incorporation of length-for-age, weight-for-length and mid-upper arm circumference in routine growth monitoring. The need for appropriate, standardised clinical records and corresponding policies to guide clinical practice and facilitate interdisciplinary communication over time became apparent.

**Conclusions:**

Clearer communication between prenatal, perinatal and postnatal health care providers, within the framework of a common understanding of growth assessment and a supportive policy environment, is a prerequisite to continuity of care and optimal health and development outcomes.

## Graphical abstract

**Figure:**
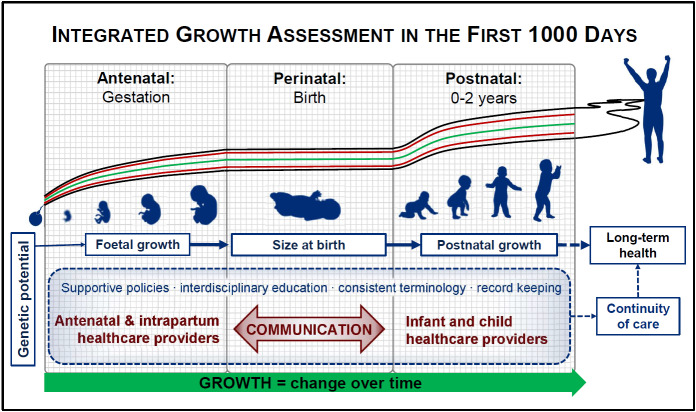

Growth assessment in the first 1000 d of life is a shared interest of all health professionals involved in the care of pregnant women, infants and children. Primary care providers, nurses, midwives, obstetricians, paediatricians and dietitians/nutrition professionals all share a common goal to support the foetus/infant to achieve its genetic potential for growth and development^(1,2)^.

In the context of growth assessment, growth refers to changes (increases/decreases) in measurable physical properties (e.g. weight, lengths and circumferences) over time^(3)^. The multi-dimensional, non-linear nature of growth necessitates sequential measurements – usually of more than one physical property – to accurately assess changes in body size, proportion and composition^(1,3,4)^. Once-off anthropometric assessment has only limited value, as any given size may be the result of consistent growth, growth faltering or excessive growth, each of which implies different health and nutritional conditions and requires different clinical management^(4)^.

Within a life-course approach, anthropometric assessment serves a purpose beyond the evaluation of current health and nutritional status; it provides summative information about past growth and predicts likely future health outcomes^(5,6)^. The first 1000 d of life (from conception to the second birthday) are particularly important as a critical window of opportunity for health promotion and disease prevention, and nutritional insults during this time can have serious short-term and life-long consequences^(7,8)^. For example, foetal growth restriction (FGR) is an important cause of potentially avoidable stillbirth^(9,10)^, size at birth may predict neonatal mortality^(11–13)^ and future growth^(14)^ and growth in infancy (as a sensitive marker for nutritional status and overall health) may predict mortality, neurodevelopment, lifetime educational achievement and future non-communicable disease risk^(7,15–17)^. Low- and middle-income countries (LMIC) remain burdened by early childhood malnutrition, and growth monitoring and promotion are a cornerstone of primary health care for children^(18–20)^. Additionally, many LMIC are experiencing a nutrition transition, with widespread chronic undernutrition complicated by increasing obesity prevalence, the so-called double burden of malnutrition. This emphasises the need for appropriate growth monitoring and timely intervention where needed^(7,17)^.

Birth is a key event in the first 1000 d of life. In physiological terms, birth represents an interruption in the growth continuum, with a temporary cessation of growth (including weight loss) as the neonate adjusts to extrauterine life. For the health care team, the perinatal period is the point of overlap between the prenatal (obstetrics, midwifery and primary antenatal care) and postnatal (paediatric, child health and primary health care) health care providers (Fig. 1). Clear communication between all these professions is essential for maintaining continuity of care and ensuring optimal outcomes for mothers, infants and young children. This necessitates that we ‘speak the same language’ across disciplines, while also staying abreast of the latest developments in the field of growth assessment. The same is true in research so that findings may be compared across studies and over time. As studies in life-course nutrition show that the consequences of poor growth may only be evident in the distant future; it is important, therefore, that at least the basic measurements lend themselves to such longitudinal analyses.

**Fig. 1:**
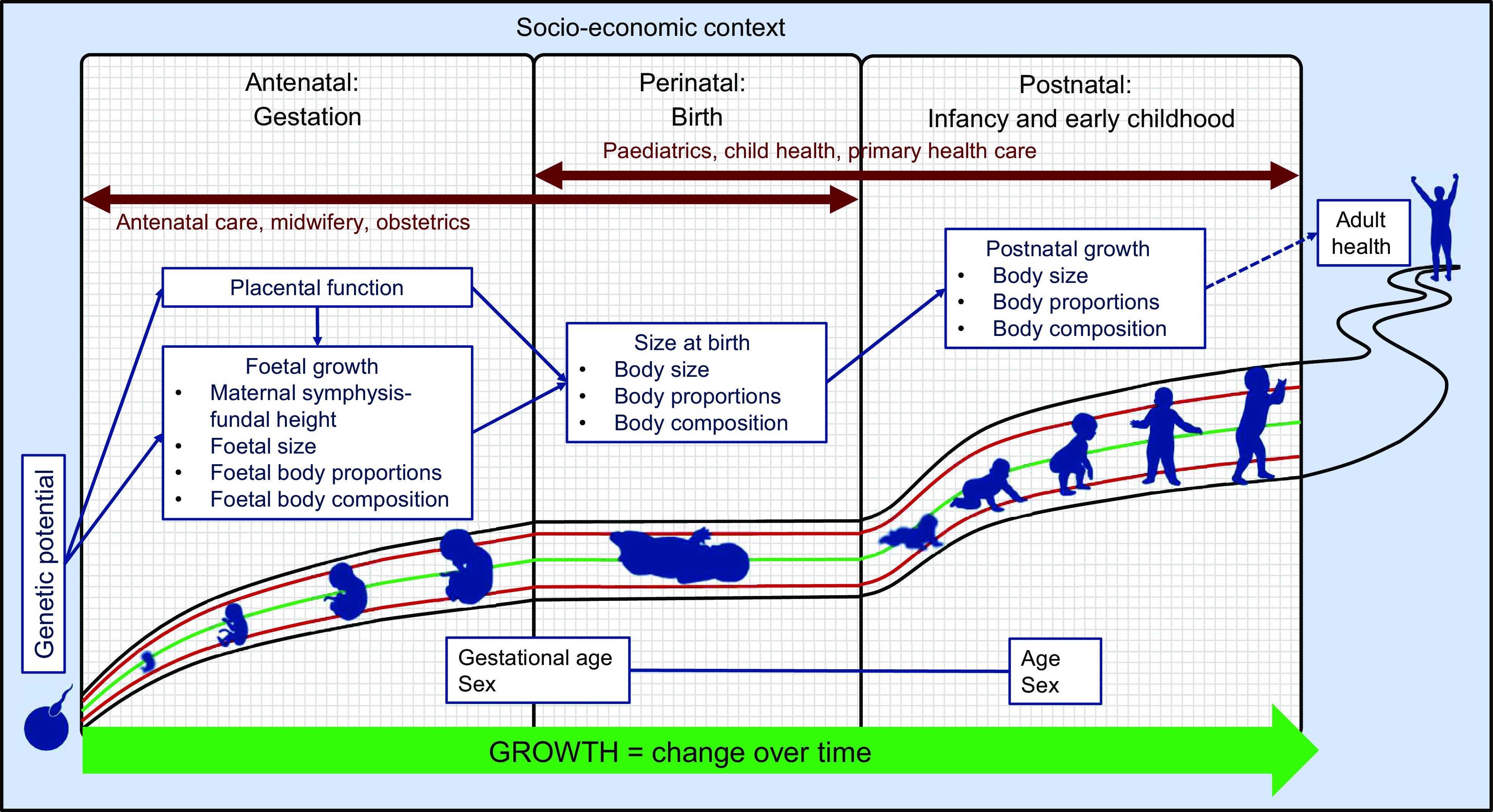
Growth assessment in the first 1000 d of life: continuity over time and across disciplines

Various factors complicate the interdisciplinary harmonisation of growth assessment in the first 1000 d. Primary among these are the fundamental differences in the established measurements, indices and indicators used by different clinical disciplines. For instance, foetal growth assessment relies on indirect measurements – maternal symphysis-fundal height, ultrasound biometric measurements and calculated estimated foetal weight – as opposed to direct measurement after birth. Discipline-specific measurements may further exacerbate this disconnect: for example, abdominal circumference is a useful measurement in the foetus but is of limited value postnatally; proportionality between weight and head circumference (HC) is of interest at birth but rarely thereafter; and nutritional assessment in infancy and childhood relies heavily on weight-for-length (WFL) or BMI which is never assessed prenatally and only rarely at birth^(21)^. This complicates communication between different disciplines. A final challenge is that policy frameworks, while encouraging interdisciplinary cooperation in principle, rarely provide any practical framework for communication across disciplines serving mother–infant dyads during different life stages^(22)^. Rather, they often perpetuate the division of prenatal, perinatal, maternal and infant healthcare by separating them at both the policy and the practical level^(23–25)^.

Addressing the communication gap between healthcare providers will require, firstly, an awareness of the overlap and discrepancies between clinical disciplines as well as an understanding of the ways in which growth is assessed by these disciplines. Clear, open communication in a common language is needed for the health care team to holistically understand the growth of the individual in their care, both in the past and in the future. Finally, the policy environment and healthcare systems should encourage and facilitate interdisciplinary communication and teamwork.

## Aim

This paper aims to contribute to evidence-based, integrated implementation of growth assessment and to contribute to the continuity of care in the first 1000 d, by the development of a unified conceptual framework that integrates measurements, indices and indicators of foetal/infant growth assessment from the time of conception until 2 years of age, with an emphasis on application in LMIC. The intention is to promote continuity of transdisciplinary health care provision, based on current scientific understanding and practice.

## Key definitions

For the sake of clarity, a distinction was made between measurements, indices and indicators (Table 1). Measurements refer to the quantification of a physical parameter. Indices combine a measurement with other measurement(s) and/or characteristic(s) such as age, gestational age (GA) and sex, in biologically meaningful ways. Indicators place indices in relation to what is expected in healthy individuals, allowing clinically useful conclusions to be drawn^(26)^.

**Table 1:** Differentiation between measurement, index and indicator in growth assessment (based on and adapted from Waterlow, 1992:213)^(26)^

Term	Definition	Examples
Measurement	The quantification of a physical parameter or property; primarily includes biometric and anthropometric data.	- Weight - Length - Abdominal circumference - Body volume (plethysmography)
Index	The combination of measurements/characteristics to allow biologically meaningful interpretation.	- Age-related e.g. weight-for-(gestational) age - Ratios e.g. BMI, foetal head circumference to abdominal circumference ratio - Calculated indices, e.g. estimated foetal weight, fat-free mass
Indicator	Tools for evaluating measurements and/or to allow for clinically meaningful judgements.	- References/standards, e.g. growth charts - Reporting systems, e.g. percentiles, z-scores, percentage of median - Classification systems, e.g. the Waterlow classification for differentiating between stunting and wasting - Cut-offs that classify and label a once-off assessment of a growth index/indices.

This paper is intended to contribute to implementation science, which may be defined as ‘an interdisciplinary body of theory, knowledge, frameworks, tools and approaches whose purpose is to strengthen implementation quality and impact’^(27)^. The topics under discussion are relevant to all professionals who assess foetal and infant growth, whether in clinical practice or for research purposes, as well as to policies governing maternal, neonatal, infant and young child healthcare provision.

## Methods

Using a multidisciplinary interactive think-tank approach over many months of regular meetings, a workgroup of researchers and clinicians with specialisation in obstetrics (*n* 4), paediatrics (*n* 2) and dietetics/nutrition (*n* 4) in consultation with a statistician, shared and debated their practice and research experience related to foetal/infant growth assessment. Weekly virtual meetings of academic staff, researchers and postgraduate students affiliated with the Research Centre were initiated in February 2021, in order to maintain contact among geographically remote affiliates, support postgraduate students and foster interdisciplinary thinking and collaboration among researchers from diverse academic backgrounds. This was considered essential in light of the transdisciplinary nature of the Research Centre’s activities. It soon became apparent that different clinical specialties within the team were approaching matters relating to foetal and infant growth from widely differing perspectives, which complicated communication among the group. Establishing a common understanding and language for matters related to growth monitoring was included as a fixed agenda item, and from these discussions, the framework presented here emerged and was formalised and refined. The discussions focussed particularly on identifying parameters that contributed meaningfully to continuous growth assessment by different clinical disciplines over the first thousand days of life. The information was grouped according to antenatal, perinatal and postnatal periods, the domains of the three clinical disciplines represented in the think-tank. The measurements, indices and indicators within each period were identified by the subject specialists in the team, based on clinical practice and evidence-based guidelines, and reviewed by all members of the team. Disagreements were resolved by discussion and referral to published research and clinical guidelines.

The information was further organised using the terminology of measurements, indices and indicators introduced by Waterlow, one of the fathers of child growth monitoring (Table 1)^(26)^. As the process advanced, recurring sub-themes emerged within the three assessment periods, namely body size and proportions, body composition and placenta-related matters (in the antenatal and perinatal periods). These were incorporated into the basic framework. The personal practice and research knowledge of the participants was supplemented and verified by published information, and evaluated for clinical applicability, particularly in resource-limited settings.

## Results

Figure 1 shows the basic conceptual framework, incorporating the somatic aspects of growth assessment within each of the three distinct assessment periods in the first 1000 d.

The genetic growth potential of the foetus/infant represents the starting point of the framework: conceptually, healthy growth is growth that achieves the genetic potential without overshooting it. However, ‘genetic potential’ is operationally difficult, as it is impossible to quantify. Recently, researchers have begun to incorporate proxies for genetic factors (e.g. ethnicity and maternal height) to develop individualised foetal growth curves and birthweight targets^(2)^. However, in practice, foetal and infant growth assessment relies on comparing the size/growth of the individual foetus/infant to the expected growth pattern described by reference growth charts^(1,4)^.

Although this framework is concerned mainly with the objective anthropometric assessment of growth, the crucial role of socio-economic factors cannot be ignored. Thus, the socio-economic context is represented as a background that encloses and underlies the framework.

Placental growth and function are included in the antenatal and perinatal phases, firstly because the placenta is derived from foetal tissue and grows in tandem with the foetus, and secondly, because of its critical role in foetal nutrition and growth. Throughout the antenatal and postnatal periods, the emphasis is on change over time, dynamically depicted by an arrow. Conversely, birth is shown as an interruption of the growth continuum, with a temporary flattening of growth curves. Anthropometric assessment at birth is commonly used as a proxy for intrauterine growth, but it provides only limited information, as an understanding of foetal growth is needed to appreciate the value and limitations of birth anthropometry.

The following sections detail the available measurements, indices and indicators in each period, followed by an integrated framework illustrating the relationship between selected growth parameters throughout the first 1000 d.

### Antenatal period: foetal growth assessment

During the antenatal period, ultrasound can be used for biometric measurements of the foetus and placenta. The measurements and functional tests, along with their associated indices and indicators, are presented in Table 2.

**Table 2:** Measurements, indices and indicators for assessment of intra-uterine growth

Measurements	Indices	Indicators
Placental size		
Placental thickness	Gestational age-related^(95)^	References/standards: no widely acknowledged references or standards available^(95)^ Cut-offs: placental thickness > 40 mm at any gestation associated with adverse maternal and foetal outcomes^(95)^
Placental function		
Doppler measurement of flow velocity in maternal-foetoplacental blood vessels - Uterine artery - Umbilical artery - Middle cerebral artery	Gestational age-related - Resistance index (RI) - Pulsatility index (PI) - Systolic/diastolic ratio - Cerebroplacental ratio (CPR = umbilical artery PI/middle cerebral artery PI)	References/standards: - INTERGROWTH-21st^(96)^ - Fetal Medicine Foundation^(97,98)^ - Pattinson graphs (Umbiflow™ device software)^(99)^ Cut-offs: - Delphi Consensus Statement for FGR* includes absent end-diastolic flow (in isolation) and umbilical/uterine artery PI > 95th percentile, CPR < 5th percentile (in combination with other indicators)^(43)^ - Umbiflow™: RI ≥ 75th percentile (Pattinson graphs)^(48,100)^
Placental grading: appearance on ultrasound	Placental maturation assessed relative to GA	References/standards: Grannum classification: grade 0–3^(101)^ Cut-offs: no objective cut-off; accelerated maturation may indicate placental insufficiency
Foetal size and body proportions		
Maternal symphysis-fundal height	GA-related	References/standards: INTERGROWTH-21st^(47)^ Cut-offs: - Most commonly used for GA determination - Repeated measures: slow growth relative to reference curve may indicate FGR^(45)^
Biometric measurements: - Long bone lengths, e.g. femur (FL), humerus. - Diameters, e.g. biparietal (BPD), occipitofrontal - Circumferences e.g. head (HC), abdomen (AC)	Individual measurements: GA-related indices Combination of measurements: (calculated) Estimated Foetal Weight (EFW) – GA-related indices	References/standards: GA-related Foetal growth charts (individual measurements and EFW): - Fetal Medicine Foundation^(32)^ - INTERGROWTH-21st^(33,34)^ - WHO Foetal Growth Charts (42) - Eunice Kennedy Shriver (NICHD)^(35)^ Cut-offs: - Delphi Consensus Statement for FGR* includes AC or EFW < 3rd percentile (in isolation) or < 10th percentile (in combination with other indicators)^(43)^ - ISUOG: 10th to 90th percentile = appropriate for GA (all measurements)^(28)^
	Proportionality - HC/AC ratio - FL/AC ratio - FL/BPD ratio	References/standards: No internationally recognised standards Cut-offs: HC/AC most used in clinical practice – median 1·2 early second trimester decreasing to 1·0 at term^(44)^
	Serial assessments: - Calculated growth velocity.^(44)^ - Individualised growth trajectories.^(2)^	References/standards: - Serial measurements can be plotted on same growth charts as individual biometric measurements - Various methods to quantify growth velocity^(44)^ (including individualised growth charts (2)), but none are widely implemented in practice Cut-offs: Delphi Consensus Statement for FGR* includes downward crossing of more than two quartiles^(43)^
Foetal body composition		
Ultrasonography: - Subcutaneous fat thickness - Organ size	Gestational age-related	Foetal body composition is not routinely assessed, and no universally acknowledged references/standards or cut-offs exist; organ size measurements may be used to assess foetal health conditions^(38)^

*Delphi Consensus Statement for Foetal Growth Restriction^(43)^:

Early FGR (GA < 32 weeks):

- Any one of: AC < 3rd centile, EFW < 3rd centile or absent end-diastolic flow in the umbilical artery, OR

- AC or EFW < 10th centile PLUS pulsatility index > 95th centile in either the umbilical or uterine artery.

Late FGR (GA ≥ 32 weeks):

- Any one of: AC < 3rd centile or EFW < 3rd centile OR

- Any two of: EFW or AC < 10th centile, AC or EFW crossing centiles by more than two quartiles on standardised growth charts, or cerebroplacental ratio < 5th centile.

Abbreviations: RI, resistance index; PI, pulsatility index; CPR, cerebroplacental ratio; INTERGROWTH-21st, International Fetal and Newborn Growth Consortium for the 21st Century; GA, gestational age; FGR, foetal growth restriction; FL, femur length; BPD, biparietal diameter; HC, head circumference; AC, abdominal circumference; EFW, estimated foetal weight; NICHD, National Institute of Child Health and Human Development; ISUOG, International Society of Ultrasound in Obstetrics and Gynecology.

Foetal measurements are assessed independently of foetal sex, but according to GA. The first important requirement to assess foetal growth, then, is an accurate GA estimate. In the absence of a certain date of conception (or last menstrual period), GA is estimated based on ultrasonographic measurements. However, this approach is less reliable after 14 weeks GA, since foetal growth becomes more variable with advancing GA^(28)^. Where ultrasound is unavailable, symphysis-fundal height measurement may be used for GA estimation, although the accuracy is inferior to ultrasound and may be challenging in women with obesity^(29–31)^.

Foetal abdominal circumference (AC), HC, femur length and biparietal diameter are commonly measured. Each of these can be assessed according to GA-specific reference charts (Table 2)^(32–36)^, with values between the 10th and 90th percentile considered appropriate for gestational age^(28)^. The same holds true for estimated foetal weight, which is calculated using the aforementioned biometric measurements. Various estimated foetal weight equations (i.e. indices) are available, including the widely used Hadlock equations^(37)^ and the newer equation from the International Fetal and Newborn Growth Consortium for the 21st Century (INTERGROWTH-21st)^(34)^. However, 10–15 % errors in estimated foetal weight *v*. actual weight are not uncommon and may be attributed to high inter- and intra-observer variability in biometric measurements, the choice of equation and the amplification of errors in single parameters when included in a calculation^(28,38–42)^.

The aim of foetal growth assessment is the detection of FGR or excessive foetal growth. Numerous diagnostic criteria (i.e. indicators) for FGR have been proposed, but the Delphi Consensus Statement for Foetal Growth Restriction (Table 2)^(43)^ is the most widely accepted. Other potentially useful indicators include biometric measurements and/or estimated foetal weight < 10th or >90th percentile for GA (indicating small-for-GA and large-for-GA, respectively)^(28)^, as well as foetal proportions and ratios (most commonly the HC/abdominal circumference ratio) as indicators of asymmetric FGR^(44)^.

Comprehensive growth assessment implies multiple measurements over time^(1,3)^, but the appropriate interpretation of serial foetal biometry remains unclear^(44)^. Various approaches have been proposed, including calculation of growth velocity, conditional percentiles, projection of expected birth weight and individualised growth charts^(2,44)^; however, none of these have been sufficiently validated for widespread incorporation into clinical practice. A minimum interval of three weeks between ultrasound assessments is recommended to minimise over-detection of foetal growth problems^(28)^.

In LMIC, ultrasound assessment is mostly limited to high-risk pregnancies. In these settings, maternal symphysis-fundal height measurement is commonly used to monitor foetal growth^(29)^. However, the sensitivity of a single symphysis-fundal height measurement for detecting small-for-GA/large-for-GA is poor except at the extremes of foetal size^(45,46)^. Nonetheless, repeated symphysis-fundal height measurements (at least 2 weeks apart), plotted on an appropriate growth chart (e.g. the INTERGROWTH-21st standard^(47)^) may be used as a first-level screening tool to identify women who require referral for ultrasound^(45,47)^.

Measures of placental function by Doppler screening may be useful to detect foetuses at risk of FGR. Doppler devices measure blood flow velocity in maternal or feto-placental blood vessels, including the umbilical, uterine and foetal mid-cerebral arteries. Various indices can be calculated (including the resistance index, pulsatility index, systolic/diastolic ratio and cerebro-placental ratio) and compared to GA-specific reference data (see Table 2), with increasing indices indicating placental dysfunction. Crucially, the low-cost, portable, easy-to-use Umbiflow™ Doppler device makes Doppler screening feasible in resource-limited settings and has value as a once-off assessment of placental function^(48)^. Optimal indicators and cut-offs for Doppler-derived indices (particularly in the absence of biometric measurements) require further investigation, although some Doppler-derived indicators are included in the Delphi Consensus Statement for FGR^(43)^.

Foetal body composition is not routinely assessed. Numerous publications describe visceral and subcutaneous fat thickness in various locations and its association with maternal diabetes mellitus, but no standards are available^(49–51)^. Likewise, measurements of foetal organ size may be used for disease detection, but do not form part of routine foetal growth monitoring^(38)^.

### Perinatal period: newborn size and body composition

For the majority of infants, birth marks the first growth assessment, as direct measurements of body size become possible. The infant and placental measurements that can be taken at birth, as well as their associated indices and indicators, are presented in Table 3.

**Table 3:** Measurements, indices and indicators that can be assessed at birth

Measurements	Indices	Indicators
Placenta		
Placental weight	Feto-placental ratio: ratio of birth weight to placental weight (BW:PW). Increases with advancing gestation (doubles from 24 weeks GA to term)^(61)^	References/standards: none, but lower BW:PW suggests impaired nutrient transfer across placenta^(61)^ Cut-offs: no universal cut-off – normal BW:PW at term = 5–7^(61)^
Body size and proportions		
Birth anthropometry: - Weight - Length - Head circumference	Gestational age-related - Weight-for-GA - Length-for-GA - HC-for-GA (Sex-specific)	References/standards: - INTERGROWTH-21st^(52,102)^ - Fenton 2013 (includes Olsen data)^(53)^ Cut-offs: Weight-for-GA^(1)^: - < 10th percentile = SGA −10th–90th percentile = AGA - > 90th percentile = LGA
	Proportionality - BW/HC ratio - Difference between BW and HC z-score - W/L ratio (GA- and sex-specific) - Ponderal index (GA-specific) - BMI (BMI; GA- and sex-specific)	References/standards: - INTERGROWTH-21st: W/L ratio-for-GA^(103)^ - Olsen 2015: BMI-for-GA^(57)^ - Landmann 2006: ponderal index-for-GA^(56)^ Cut-offs: - BW/HC ratio < 90 (proposed, unvalidated)^(58)^ - HCZ-BWZ > 1 (proposed, unvalidated)^(59)^ - PI < 2 or < 10th percentile for GA^(13,55)^
Body composition		
Body volume (by air-displacement plethysmography)	Body density à FM, FFM à related to weight: %FM, %FFM à related to length: FMI, FFMI (Sex- and GA-specific)	References/standards: - INTERGROWTH-21st: (36–42 weeks GA) – FM, %FM, FFM^(103)^ - Norris 2019 (30–41 + 6 weeks GA) – FM, %FM, FFM^(104)^ Cut-offs: None established

Abbreviations: BW, birth weight; PW, placental weight; GA, gestational age; HC, head circumference; INTERGROWTH-21st, International Fetal and Newborn Growth Consortium for the 21st Century; SGA, small for gestational age; AGA, appropriate for gestational age; LGA, large for gestational age; W, weight; L, length; HCZ, head circumference-for-GA z-score; BWZ, birth weight-for-GA z-score; PI, ponderal index FM, fat mass; FFM, fat-free mass; FMI, fat mass index; FFMI, fat-free mass index.

Meaningful interpretation of newborn size relies on sex- and GA-specific reference data (e.g the INTERGROWTH-21st Newborn Size Standards^(52)^ and the Fenton 2013 growth chart^(53)^). The 10th and 90th percentiles of birth weight-for-GA are commonly used to distinguish small, appropriate and large for GA infants^(1)^. However, birth anthropometry only gives a summative snapshot of foetal growth, without any indication of the preceding foetal growth trajectory. Thus, true FGR or excessive foetal growth may be missed. For example, a neonate with birth weight < 10th percentile may simply be constitutionally small, yet growing consistently (i.e. achieving its genetic growth potential), whereas a foetus with faltering growth may remain above the 10th percentile at birth^(1,54)^. This highlights the crucial importance of communication between pre- and postnatal healthcare providers: measurements taken during pregnancy (e.g. serial ultrasound biometry or umbilical artery Doppler) can help to identify truly growth-restricted neonates who are at risk of adverse outcomes and guide paediatric healthcare providers’ expectation for appropriate postnatal growth.

In the absence of antenatal measurements, the proportionality (or symmetry) of the neonate can provide clues about foetal growth. An infant is considered proportional/symmetrical if the GA-related z-scores for weight, length and HC are similar; conversely, if the z-score for weight is markedly lower than that of length and/or HC, the neonate is considered asymmetrically growth restricted. Asymmetrical growth restriction is believed to result from cranial redistribution of foetal circulation due to placental insufficiency, maintaining brain growth at the cost of somatic growth^(13,55)^. Various attempts have been made to mathematically quantify the relationship between weight and length (including weight-length ratio^(56)^, BMI-for-GA^(57)^ and ponderal index^(13,56)^) or weight and HC (including BW/HC ratio^(58)^ and the difference between HC and BW z-scores^(59)^), but none of the related cut-off values have been sufficiently validated for adoption into routine clinical practice. Likewise, it is not yet clear which, if any, of these indices and indicators have superior predictive value for short- and long-term adverse outcomes.

The placenta is routinely weighed at birth, but standardised procedures and consensus on indicators of abnormality are lacking. The birth weight to placental weight ratio (BW:PW; also called the foeto-placental ratio) has been shown to correlate with foetal abdominal circumference growth velocity and Doppler indices of placental function; as such, a low BW:PW ratio may suggest a history of placental insufficiency and FGR^(60)^. The BW:PW ratio increases with advancing gestation, doubling from 24 to 38–40 weeks to reach a ratio of 5–7:1 at term, but a definite cut-off for abnormality remains elusive^(61)^.

Body composition at birth can be assessed using air-displacement plethysmography (ADP, e.g. using the PEAPOD™ device), which measures body volume and calculates body density, fat mass (FM) and fat-free mass (FFM). Further indices can be calculated relative to total body weight (%FM and %FFM) or length (fat mass index and fat-free mass index – that is, FM or FFM (in kg) divided by length (in m) squared). The interpretation of these indices is linked to infant sex and GA. Various reference charts are available (see Table 3), but as yet no cut-offs have been established for body composition indicators.

### Postnatal period: growth in infancy and early childhood

Routine growth monitoring has long been one of the cornerstones of primary health care provision for infants and children^(18)^. The various measurements, indices and indicators for assessing growth in the first 2 years after birth are shown in Table 4.

**Table 4:** Measurements, indices and indicators for assessment of growth in infancy and early childhood

Measurements	Indices	Indicators
Body size and proportions		
Weight Length	Once-off assessment - Weight-for-age - Length-for-age - Weight-for-length - BMI-for-age (Sex-specific) (GA-specific and/or age-corrected for preterm infants)	References/standards: - Preterm infants: Fenton 2013^(53)^, INTERGROWTH-21st Preterm Postnatal Growth Standards^(90)^ - WHO MGRS Growth Standards^(66)^ - Population- and disease/condition-specific growth charts Cut-offs: WHO MGRS Growth Standards^(62,105)^ - WFA z-score <–2 = underweight (<–3 = severe) - WFL z-score <–2 = wasting (<–3 = severe) - LFA z-score <–2 = stunting (<–3 = severe) - BMI-for-age z-score >+2: = overweight WHO/UNICEF diagnostic criteria for moderate/severe acute malnutrition (MAM/SAM) (6–59 months) include WFL (with MUAC and clinical assessment)^(65)^ - WFL z-score <–2 = MAM, <–3 = SAM
	Serial assessments - Weight velocity (e.g. g/interval) - Length velocity (e.g. cm/interval) (Sex-specific)	References/standards: WHO MGRS Growth Standards^(71)^ Cut-offs: None established
Head circumference	Once-off assessment HC-for-age (Sex-specific)	References/standards: WHO MGRS Growth Standards^(67)^ Cut-offs: HC z-score <–2 or >+2 warrants investigation
	Serial assessments: HC velocity (cm/interval) (Sex-specific)	References/standards: WHO MGRS Growth Standards^(71)^ Cut-offs: None established
MUAC	MUAC-for-age (Sex-specific)	References/standards: WHO MGRS Growth Standards^(67)^ Cut-offs: WHO/UNICEF diagnostic criteria for moderate/severe acute malnutrition (MAM/SAM) (6–59 months) include MUAC (with WFL and clinical assessment)^(65)^: - MUAC < 115 mm = SAM −115mm ≤MUAC < 125 mm = MAM
Skinfolds: triceps, subscapular	- TSF-for-age - SSF-for-age (Sex-specific)	References/standards: WHO MGRS Growth Standards^(67)^ Cut-offs: None established
Body composition		
Body volume (plethysmography)	Body density à FM, FFM à in relation to weight: %FM, %FFM à in relation to length: FMI, FFMI (Sex- and GA-specific)	References/standards: - Norris 2019 (1–27 weeks) - FM, %FM, FFM^(104)^ - De Fluiter 2020 (0–6 months) - FM, %FM, FMI, FFM, FFMI^(85)^ Cut-offs: None established
Stable isotope concentration (dilution)	Total body water à FM, FFM à related to weight: %FM, %FFM à related to length: FMI, FFMI (Sex- and age-specific)	References/standards: - Wells 2020 (6 weeks to 5 years) - TBW, FM, FMI, FFM, FFMI^(106)^ Cut-offs: None established
X-ray attenuation (DEXA)	FFM (differentiated into bone mineral content, cell mass (water, protein)), FM. à FM, FFM à related to weight: %FM, %FFM à related to length: FMI, FFMI (Sex- and age-specific)	References/standards: - De Fluiter 2020 (6–24 months) - FM, %FM, FMI, FFM, FFMI^(85)^ Cut-offs: None established

Abbreviations: GA = gestational age; INTERGROWTH-21st, International Fetal and Newborn Growth Consortium for the 21st Century; WHO, World Health Organization; MGRS, Multicentre Growth Reference Study; WFA, weight-for-age; LFA, length-for-age; WFL, weight-for-length; MUAC, mid-upper arm circumference; UNICEF, United Nations Children’s Fund; MAM, moderate acute malnutrition; SAM, severe acute malnutrition; HC, head circumference; TSF, triceps skinfold; SSF, subscapular skinfold; FM, fat mass; FFM, fat-free mass; FMI, fat mass index; FFMI, fat-free mass index; DEXA, dual-energy X-ray absorptiometry.

Anthropometric and body composition parameters are interpreted according to the infant/child’s sex and age (with age correction for preterm infants born at < 37 weeks GA). Weight, length, HC and mid-upper arm circumference (MUAC) are commonly measured, while triceps skinfold and subscapular skinfold are technically much more challenging and much less commonly used. Any of these measurements can be interpreted as a sex-specific index-for-age (i.e. weight-for-age, length-for-age, HC-for-age, MUAC-for-age, triceps skinfold-for-age and subscapular skinfold-for-age), while weight can also be interpreted in relation to length, e.g. WFL or BMI (weight (in kg) divided by length (in m) squared), interpreted as BMI-for-age. This is particularly important in LMIC, where the high prevalence of stunting complicates the interpretation of simple weight-for-age.

Growth charts remain the cornerstone of interpreting growth indices in childhood^(62)^. WHO Multicentre Growth Reference Study (MGRS) Growth Standards are intended as a single, global growth standard by which children of all nationalities and ethnicities can be assessed^(63,64)^, although some countries continue to use population-specific growth charts^(20)^. Growth standards can be used to determine percentile positions and/or calculate z-scores or to identify the median of a given growth index. Z-score values between –2 and +2 are considered normal, although a small proportion (< 5 %) of a normally distributed population could be expected to fall outside these limits.

Different indicators and classification systems exist for the diagnosis of malnutrition, based on weight-for-age, WFL and length-for-age, and using either z-scores or the measured value as a percentage of the median (see Table 4). Additionally, MUAC is used as such to identify moderate and severe acute malnutrition in children aged 6 months to < 5 years^(65)^. MUAC-for-age charts are available^(66)^, but the appropriate interpretation of z-scores is not clear. The interpretation of BMI-for-age to identify overweight in young children is controversial, but the rise of obesity among children even in LMIC cannot be ignored^(7,17)^. Skinfold measurements may be used as a rough indicator of adiposity, and reference data for triceps skinfold and subscapular skinfold are available (WHO-MGRS Growth Standards^(67)^, but the appropriate interpretation remains unclear. Additionally, skinfolds are technically challenging to measure, and intra-observer measurement reliability tends to be poor^(68)^.

Anthropometric status (WFL z-score, MUAC) at a single time point may be used to classify moderate and severe acute malnutrition in children aged 6–59 months. Additionally, the presence of bilateral nutritional oedema and/or severe wasting is diagnostic of severe acute malnutrition regardless of anthropometric status^(65)^. Indicators of malnutrition based on the percentage of the median of older reference data have become obsolete in light of the WHO MGRS growth standard, and their routine use is no longer recommended. These include the Waterlow classification^(69)^ (used to distinguish between wasting (low WFL) and stunting (low length-for-age)) and the Wellcome classification^(26)^ (used to distinguish between underweight, wasting, kwashiorkor and marasmic kwashiorkor, on the basis of weight-for-age and the presence of oedema).

Growth, as stated earlier, refers to change over time; thus, serial interpretation of anthropometric indices is imperative^(62)^. Healthy growth is characterised by growth indices maintaining approximately constant z-scores over time, with some intra-individual variation^(62)^. It is unclear what degree of deviation from an individual’s expected z-score trajectory should be considered problematic^(62,70)^. Nonetheless, accurately plotting of consecutive measurements on a suitable growth chart, with consideration of multiple indices, remains the best available method to identify growth faltering or excessive growth^(4,62,70)^. The WHO-MGRS Growth Standards include growth velocity standards for weight, length and HC^(71)^ but these indices are challenging to use and interpret.

Growth assessment in preterm-born infants is less straightforward. There are several controversies, including the type of growth charts to use (charts based on longitudinally collected data, such as the INTERGROWTH-21st Postnatal Growth Standards for Preterm Infants, *v*. charts based on cross-sectional birth data, such as the Fenton 2013 Growth Charts), the most desirable growth trajectory (regaining the birth weight z-score, maintaining the z-score achieved after the initial weight loss, or various methods for calculating growth velocity), and the desirability of catch-up growth and speed thereof^(4,72–77)^. Similarly, growth assessment in infants and children with certain chronic conditions can be challenging. Length measurement may be impossible in infants/children with neurological impairment, necessitating the use of proxy measurements such as limb segments and circumferences^(78)^. Furthermore, standard growth charts may not be applicable in conditions where ‘normal’ growth cannot reasonably be expected. Specialised growth charts are available for several clinical conditions, including (but not limited to) cerebral palsy^(79)^, Down Syndrome^(80)^, Turner Syndrome^(81)^, Prader–Willi syndrome^(82)^ and Noonan Syndrome^(83)^, but their use is not universally accepted, particularly in infancy and early childhood^(78)^. Body composition assessment may be of value in these populations, although the appropriate indicators of malnutrition remain unclear^(78)^.

Body composition can be assessed by various methods in the first two postnatal years. Although uncommon in clinical practice, research settings may use ADP, stable isotope dilution and dual-energy X-ray absorptiometry^(68)^. Body volume is measured by ADP and combined with weight to calculate body density, from which FM and FFM can be calculated^(68,84)^. The PEAPOD™ ADP device is only suitable for infants up to 10 kg^(84,85)^, which some infants may reach already at 6–8 months, although smaller infants may remain under 10 kg up to 2 years of age or beyond^(66)^. The BODPOD™ with paediatric attachment can be used for infants from 12 kg, but is only validated from 2 years of age^(86)^. Stable isotope dilution may be used to assess body composition in infants and children of any age but may be impractical in neonates^(68,87)^. The method involves administering a known dose of a stable isotope (e.g. deuterium-containing water), allowing it to equilibrate in the body and measuring its concentration in saliva or urine. This allows for the calculation of total body water mass and, by extension, FFM and FM^(87)^. The final method, dual-energy X-ray absorptiometry, estimates body composition from the measured attenuation of X-rays through the body^(68)^. Unlike the previous two methods, dual-energy X-ray absorptiometry can further distinguish between bone and soft tissue (water and protein). The high cost limits the availability of dual-energy X-ray absorptiometry, and repeated X-ray exposure may not be desirable^(68)^. Reference data have been published for each of these methods (Table 4). Bioelectrical impedance analysis has been proposed as potentially useful in infants, but at this time none of the available predictive equations for the calculation of body composition parameters have been sufficiently validated to be recommended for clinical or research use, as found in a recent (2021) systematic review^(88)^. It should be emphasised that body composition data obtained using different methods are not interchangeable^(68,89)^, so it is important to use reference data compiled using the same methodology. No cut-off values for indicators of body composition abnormality have been established.

### Integrated framework

**Fig. 2:**
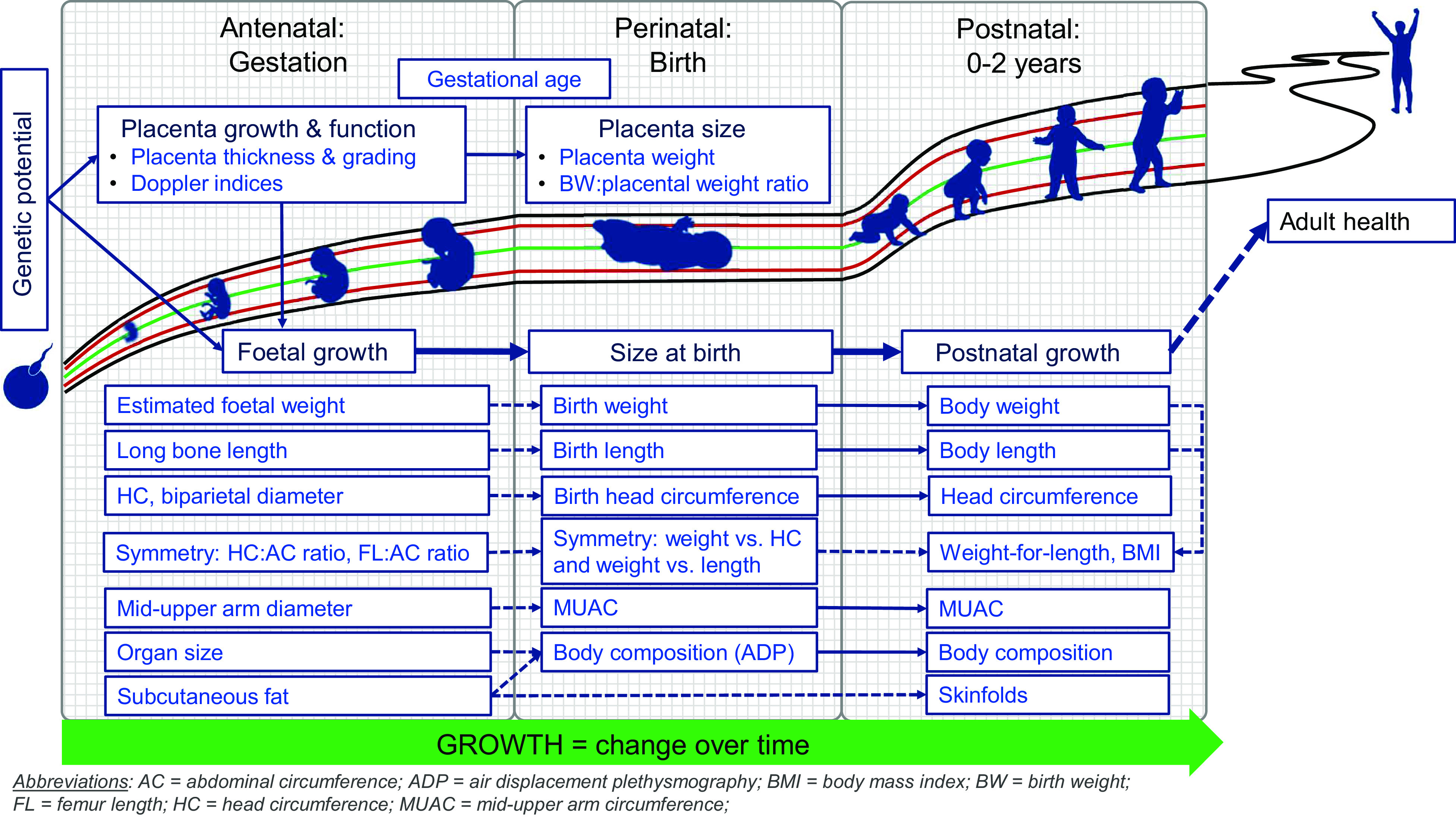
Integration of growth parameters throughout the first 1000 d of life

The preceding discussion identifies numerous measurements, indices and indicators for assessing growth in the first 1000 d of life. Integrating these disparate parameters into a single, unified framework implies identifying those parameters that can meaningfully be compared across the antenatal, perinatal and postnatal periods. The relationship between selected measurements and indices is illustrated in Fig. 2, with solid arrows indicating directly comparable parameters and dashed arrows indicating parameters that are related but not identical. The transition between prenatal and postnatal growth assessment is necessarily somewhat disjointed due to the indirect nature of foetal biometry.

## Discussion and recommendations

Growth assessment in the first 1000 d of life involves numerous clinical disciplines, measurements, indices and indicators. Growth assessment is inextricably linked to a life-course approach: current growth status results from earlier growth patterns and guides future clinical management. Unfortunately, limited contact between pre- and postnatal healthcare providers undermines interdisciplinary communication and continuity of care. This paper proposes an integrated, interdisciplinary framework for growth assessment in the first 1000 d of life, with the aim of promoting a common understanding among all clinical disciplines involved in this critical period.

Developing a common language among professions as diverse as obstetrics, midwifery, paediatrics, nursing and dietetics/nutrition is no small feat. A shared understanding of measurements and indices and the relationship between them (as shown in Fig. 2) is an important first step. Of course, not all possible measurements, indices and indicators can (or need to) be included in a unified framework, but those parameters that may meaningfully predict future outcomes should be understood by and communicated to all. To this end, differences in terminology, measurement methods, reference data, reporting systems and indices and indicators of interest need to be identified and, where possible, harmonised. For example, in paediatrics, the use of z-scores has become ubiquitous due to their mathematical and statistical utility, whilst percentiles are still preferred in many obstetric settings. Indeed, until recently, no z-score reference data were available for foetal growth indices, but presently the INTERGROWTH-21st Fetal Growth Standards^(33,34)^ and Fetal Medicine Foundation’s Fetal Growth calculator^(32)^ both include z-scores. Furthermore, the INTERGROWTH-21st Growth Standards and the Fenton 2013 Growth Chart all demonstrate good continuity with the WHO MGRS Growth Standards, further facilitating the integration of pre- and postnatal growth assessment^(53,90–92)^. Thus, the tools for integration of growth assessment from conception through childhood are there; it is up to us – the clinical and research communities – to embrace these opportunities and optimise patient care and research reporting. Selecting the best set of tools to facilitate such integration will require careful consideration of the scientific merits and drawbacks of each reference. The debate surrounding the optimal growth chart for preterm infants is a good example: despite the conceptual coherence and strict individual-level inclusion criteria of the INTERGROWTH-21st Postnatal Growth Standards for Preterm Infants, the limited sample size at postmenstrual ages < 36 weeks is a serious concern^(72)^. For this reason, the American Academy of Paediatrics recommend the use of intrauterine (i.e. birthweight-derived) charts for monitoring the postnatal growth of preterm infants^(93)^. Evidence of the superiority of one growth chart over the other, particularly in ethnically diverse LMIC populations, is still lacking.

Three additional practical considerations underlie the successful integration of growth assessment across the first 1000 d. Firstly, measurements must be taken accurately, which requires functional equipment and adequately skilled and motivated measurers. This also implies standardised measurement techniques, e.g. of length measurements at birth and during infancy. Secondly, accurate assessment and documentation of GA is crucial, owing to the non-linearity of growth in the first 1000 d. And finally, measured values must be documented and made available to all members of the health care team. In industrialised countries, universally accessible electronic health records can facilitate this. Where such infrastructure may not be available, a patient-held document (such as the child’s vaccination record) may fulfil the same role.

The measurements, indices and indicators outlined in this paper represent an ideal. However, some of the mentioned equipment and skills may not be available in LMIC and other resource-limited settings. Nonetheless, there are feasible, accessible practices that can be incorporated to improve growth assessment and contribute meaningfully to patient care outcomes. In the prenatal period, routine screening with a low-cost Doppler device (e.g. the Umbiflow™ device) has proven valuable for detecting foetuses at risk of FGR and stillbirth^(48,94)^. At birth, accurate assessment of length and HC, and standardised weighing of the placenta (with calculation of BW:PW ratio), may provide important information about foetal growth. During infancy and childhood, routine assessment of length-for-age, WFL and MUAC can provide valuable information about health and nutrition status that will be missed if only weight-for-age is assessed.

**Fig. 3:**
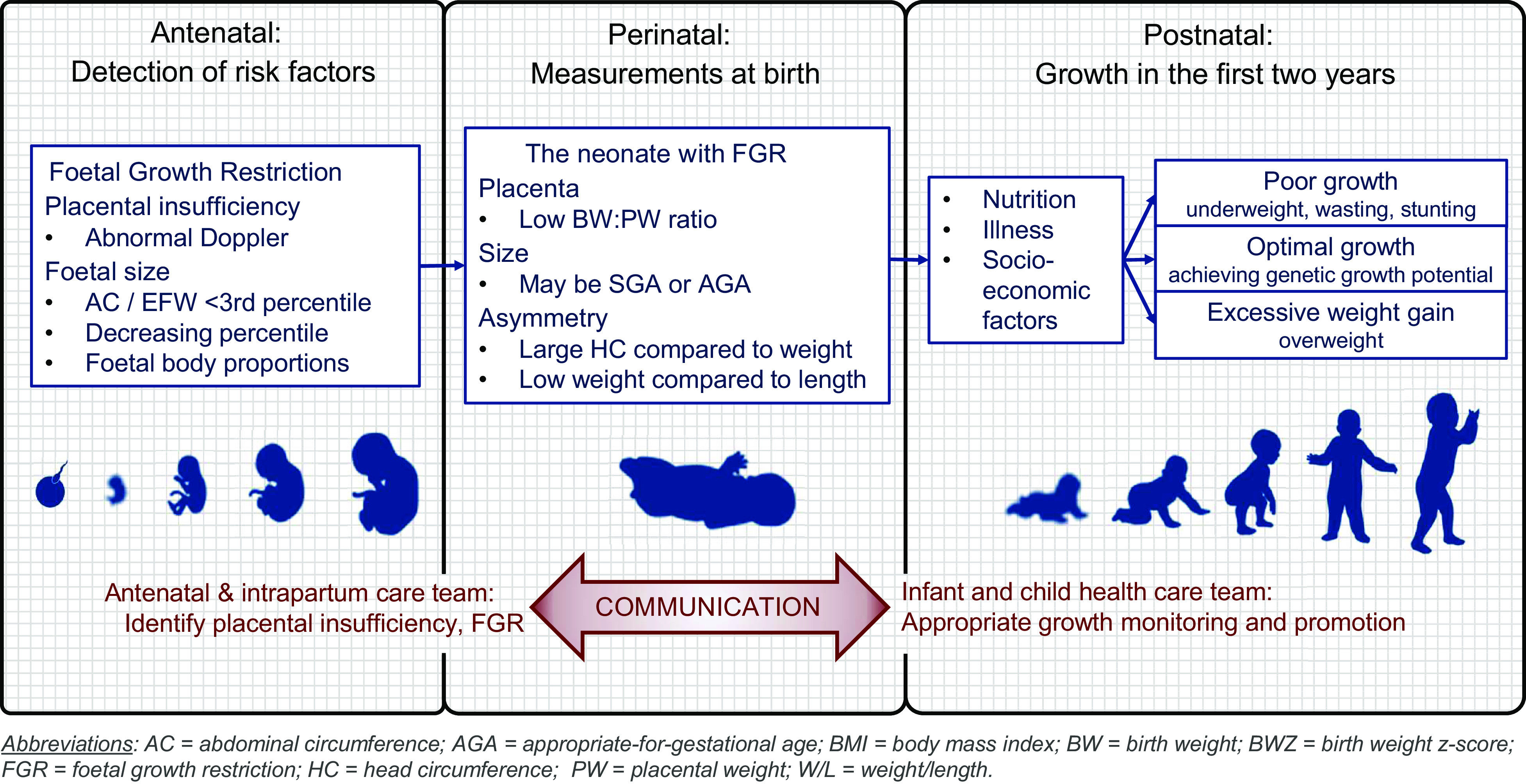
Birth as link between antenatal and postnatal health care teams, in the context of identifying and managing the neonate with foetal growth restriction (FGR)

Figure 3 places this theoretical discussion into a practical context, showing how clear communication and information sharing between the antenatal and postnatal care teams can help identify neonates with FGR, who may be at increased risk of growth anomalies, and guide appropriate growth monitoring and promotion in infancy and childhood. Crucially, this information allows postnatal care providers to set appropriate growth targets for postnatal growth and tailor nutrition interventions accordingly. Nutrition, acute/chronic illness and socio-economic factors are highlighted as important determinants of growth; these represent potential areas for intervention to optimise growth and, by extension, long-term health. It also underscores the fact that some factors contributing to poor/excessive growth are systemic and not under the control of the individual. The purpose of clinical labelling is to achieve a concise common language among those involved in health promotion, malnutrition prevention and clinical care. Stigmatisation and blaming of the child and caregiver should be avoided at all cost. Using non-judgemental ‘people-first’ language – for example, using the term ‘child with obesity’ or ‘child with a BMI in the obese category’ rather than ‘obese child’ – can further help to mitigate possible negative effects of clinical labelling.

### Policy and programmatic implications

Any change in the *status quo* requires a supportive policy environment. In a public healthcare system that uses standardised clinical documents and practice guidelines, the following is recommended:

- Incorporating Doppler screening as a test for placental function in basic antenatal care services and recording these results in the child’s health record and vaccination card at birth;
- Standardising measurement techniques of birth anthropometry, particularly length measurement;
- Standardising methods for weighing the placenta, and including calculation of BW:PW ratio;
- Incorporating newborn growth charts for birth weight, HC and length in maternity and child health care records;
- Including the measurement of length and the assessment of WFL and/or BMI-for-age in routine growth monitoring; and
- Ensuring that all policies and practices relating to health care worker education, clinical practice and clinical record keeping foster and support the integration of health care across disciplines and over time.

Each of these recommendations requires investment in equipment and training, but the benefit to the lives of infants and young children is likely to be substantial. The political will of policy makers, integrated record-keeping systems, the willingness of health care practitioners to put child well-being above disciplinary advancement, fostering teamwork in professional education and ongoing validation of growth assessment are at the foundation of achieving each child’s genetic potential in the first 1000 d.

### Recommendations for research and practice

This framework is not presented as a conclusive standard, but rather as a proposal to prompt discussion, collaboration and research. The long-term usefulness of some indices and indicators (e.g. the various proposed indicators of asymmetry at birth) in predicting short- and long-term adverse outcomes presents an important research opportunity. For this reason, we also recommend that basic measurements (e.g. accurate birth weight, length and HC) always be carefully done and recorded, as the emergence of new indices/indicators can then potentially allow for re-analysis of existing datasets. Research into novel measurements and indices is ongoing, yet even well-established indices still lack agreement in terms of indicators and cut-offs. Research in these fields should focus on identifying indicators that can usefully predict important outcomes, including mortality, morbidity, growth and neurodevelopment. The validity, predictive value and optimal cut-offs of proxy indicators to replace ultrasonography (such as Doppler screening and measurement of the placenta at birth) particularly require investigation. Finally, interdisciplinary approaches should be integrated into pre-service education and continuing professional development initiatives.

## Conclusion

Growth occurs on a continuum from conception throughout infancy and childhood, with events in the first 1000 d of life known to have life-long effects. It is crucial, therefore, that clinicians providing care to mothers, infants and young children in different life stages should be able to clearly communicate about common goals and concerns. This paper presents a framework to act as a starting point for such an integrated approach and also highlights areas where further research and policy initiatives are required. Clear communication, a collaborative approach and strong policy-level support will be needed to ensure continuity of care throughout the first 1000 d of life and ultimately promote optimal health and developmental outcomes.
